# Gut microbiota in patients with sarcopenia: a systematic review and meta-analysis

**DOI:** 10.3389/fmicb.2025.1513253

**Published:** 2025-01-22

**Authors:** Guangning Wang, Yujie Li, Huisong Liu, Xinjuan Yu

**Affiliations:** 1 Department of Critical Care Medicine, Qingdao Hospital, University of Health and Rehabilitation Sciences, Qingdao, China; 2 Reproductive Medicine Center, Women and Children’s Hospital, Qingdao University, Qingdao, China; 3 Department of Nursing, Qingdao Hospital, University of Health and Rehabilitation Sciences, Qingdao, China; 4 Department of Clinical Research Center, Qingdao Hospital, University of Health and Rehabilitation Sciences, Qingdao, China

**Keywords:** gut microbiota, sarcopenia, musculoskeletal diseases, effects, meta-analysis

## Abstract

**Background:**

Intestinal dysbiosis was considered a pivotal pathological mechanism underlying sarcopenia. Despite the fervor surrounding research in this domain, substantial controversy persists regarding the obtained outcomes.

**Objective:**

To systematically summarized the disparities in gut microbiota composition between the group afflicted by sarcopenia and non-sarcopenia controls.

**Methods:**

PubMed, Medline, CINAHL, EMBASE, Scopus, Web of Science and Google Scholer, Cochrane Library and gray literature sources were systematically searched for in randomized controlled trials. Meta-analysis and random-effects meta-regression were conducted using Rev. Man 5.3. Overall effect was measured using Hedges’s g and determined using Z-statistics. Cochran’s *Q* test and *I*^2^ were used to investigate heterogeneity. The Newcastle-Ottawa Scale was used to assess overall quality of evidence.

**Results:**

Ten studies, including 421 cases of sarcopenia and 1,642 cases of controls, were included in the meta-analysis. Patients with sarcopenia showed significantly reduced gut microbiota in α diversity, and β diversity was significantly different in 8/9 of included studies. We also found more abundance of phylum Proteobacteria and genus *Escherichia-Shigella*, and less abundance of phylum Firmicutes and genus *Faecalibacterium*, *Prevotella 9*, *Blautia* in the sarcopenia group.

**Conclusion:**

The gut microbiota composition in patients with sarcopenia has undergone alterations, serving as a fundamental reference for further investigation into the potential pathogenic mechanisms and treatment strategies for sarcopenia.

## 1 Introduction

Sarcopenia is a prevalent age-related skeletal muscle disorder characterized by the progressive loss of muscle mass and decline in muscular strength. Sarcopenia has an incidence of 36% in individuals <60 years, and 27% in individuals ≥60 years, the prevalence of severe sarcopenia was as high as 9%, and this percentage continues to increase with age (Petermann-Rocha et al., 2022). The number of people with sarcopenia is predicted to increase to 1.2 billion by 2025 and double by 2050 (Almohaisen et al., 2022). It is one of the leading health issues in the older adults, and it increases disability risk, falls as well as injuries related to falls, hospitalization, limitation of independence, and mortality, It has a certain burden on the social medical system (Cruz-Jentoft et al., 2019; Sayer and Cruz-Jentoft, 2022). Age-related mechanisms that contribute to the onset of sarcopenia encompass inflammation, immunosenescence, anabolic resistance, reduced levels of physical activity, and heightened oxidative stress (Chen et al., 2020; Cho et al., 2022).

The gut microbiota exerts a pivotal role in the aging process by regulating energy balance, metabolism, and inflammation to impact the progression of sarcopenia (Papadopoulou, 2020; Ling et al., 2022). The gut microbiota is recognized as an overlooked endocrine organ, exerting regulatory control over the host’s homeostasis through the fermentation of undigested food in the colon, thereby generating a wide array of bioactive molecules (Fan et al., 2023). For instance, short-chain fatty acids (SCFAs), such as acetate, propionate, and butyrate derived from dietary fiber, have demonstrated favorable effects on the host by enhancing skeletal muscle growth. The recently proposed concept of the gut-muscle axis suggests a potential correlation between gut microbiota and the quality and functionality of skeletal muscle (Liao et al., 2020). Yan et al. showed that the diversity and richness of gut microbiota were lower in sarcopenia patients than in controls. Among them, a decrease in the ratio of Prevotella to Bacteroidetes (P/B) and a decrease in the abundance of Coprococcus and Lachnospiraceae were significant indicators. Prevotella and Bacteroidetes are involved in dietary fiber fermentation and the production of short-chain fatty acids (SCFAs), which are essential for the maintenance of muscle mass and function. A lower P/B ratio indicates a reduced capacity for SCFA production, which may negatively affect muscle health. Furthermore, the reduction in the quantity of Faecalibacterium and Lachnospiraceae, which are the main producers of short-chain fatty acids, is closely related to the decrease in short-chain fatty acid levels, which may further lead to muscle atrophy and weakened muscle strength. In view of this, monitoring these specific bacterial markers can provide us with early warning signals of sarcopenia, enabling us to take timely intervention measures (Yan et al., 2023). Research into the association between gut microbiota and muscle frailty in elderly populations has demonstrated that the composition of intestinal flora undergoes significant alterations in sarcopenia patients. Specifically, the relative abundance of Lactobacillus, Bacteroides, and Prevotella decreases, whereas that of Leminoxella increases markedly (Grosicki et al., 2018). Through pyrosequencing analysis of 16S rRNA, it was found that the quantities of Ruminococcus and Brucella in the intestines of sarcopenia patients decreased significantly, while the abundance of *Escherichia coli* increased (Grosicki et al., 2018). It is worth noting that as people age, the composition of the human intestinal flora changes due to muscle weakness (Dao et al., 2020). Through the study of 35 community residents over 70 years old (including 18 sarcopenia patients and 17 healthy controls), it was shown that the quantities of Helicobacter and Ruminococcus in the intestines of sarcopenia patients increased, while the quantities of Pasteurellaceae and Christensenellaceae decreased (Wang X. et al., 2022; Wang Y. et al., 2022). In addition, the serum aspartate concentration was higher in sarcopenia patients, while the circulating levels of threonine and macrophage inflammatory protein 1α were lower.

In recent years, more research has focused on the relationship between sarcopenia and gut microbiota composition. At the genus level, Prevotella and Lacococcus faecalis have been identified as key markers and studies have shown their significantly reduced abundance in the sarcopenia population (Kang et al., 2021). At the family level, Trichospiraceae and Ruminoccaceae have received much attention for their important role in intestinal health and metabolism in patients with sarcopenia (Zhou et al., 2023). At the phylum level, the relative abundance of Firmicutes and Bacteroidetes was significantly decreased in patients with sarcopenia (Kang et al., 2021). The above taxa are believed to play an important role in the development of sarcopenia by affecting the inflammatory response and energy satisfaction.

In recent years, the emergence of high-throughput sequencing technologies such as 16S rRNA and metagenomics has sparked increasing interest in exploring the potential role of gut microbiota in the pathogenesis of muscular dystrophy (Wang X. et al., 2022; Wang Y. et al., 2022). However, the existing research results of 16S rRNA sequencing are inconsistent, and there is a lack of systematic summary, making it difficult to provide clear reference basis for the prevention and clinical treatment of sarcopenia. Therefore, this study aims to explore the differences in diversity and richness of the gut microbiota between sarcopenia and non-sarcopenia populations through systematic review and meta-analysis, in order to clarify the potential role of GM and its metabolites in the pathogenesis of sarcopenia, and provide new theoretical basis and practical methods for the prevention and treatment of sarcopenia.

## 2 Methods

### 2.1 Literature search

The present systematic review and meta-analysis was conducted in accordance with the guidelines provided by the Preferred Reporting Items for Systematic Reviews and Meta-Analyses (PRISMA) (Page et al., 2021). Full-text original research articles on sarcopenia individual and non-sarcopenia individual were identified in a search of the PubMed, Medline, CINAHL, EMBASE, Scopus, Web of Science and Google Scholer, and Cochrane Library and gray literature sources, from the establishment of the database until February 2024. A combination of subject words and corresponding free words were used for the search, including “sarcopenia” OR “Gut-Muscle axis” OR “skeletal muscle,” “Muscle mass,” “microbiota,” “gut microbiota,” “microbiome,” “metabolomic,” “intestinal flora.” The study protocol was registered with PROSPERO (CRD42024523222).

### 2.2 Eligibility criteria

The two authors (WGN and LYJ) independently conducted a thorough examination and selection of the complete texts that satisfy the specified criteria: (1) These articles are peer-reviewed and written in English; (2) They compare the diversity and abundance of gut microbiota between patients with sarcopenia and healthy or non-sarcopenia control groups; (3) The gut microbiota is derived from fecal samples; (4) Participants include adults aged 18 years and above, excluding studies focused on children as their microbial composition is less stable during development and cannot be compared to adults; (5) Sufficient statistical data should be provided, such as mean, standard deviation, interquartile range, p-value, maximum value, minimum value, etc., to estimate effect size; (6) Alpha diversity indices that can be correctly collected should be presented in the article or Supplementary material (e.g., tables, box plots, bar graphs). Case reports, systematic reviews, and animal studies are excluded.

### 2.3 Outcome measures

The findings encompass the diversity of gut microbiota (including α-diversity and β-diversity) as well as variations in gut microbiota abundance between individuals with muscular dystrophy and healthy or non-muscular dystrophy control groups.

### 2.4 Data extraction and quality assessment

Two researchers (WGN and LYJ) independently screened eligible studies and excluded articles that did not meet the inclusion criteria. The following data were collected: article title and publication year, country of origin, age range of participants, gender distribution, sample size, method used for microbial assessment, region targeted for 16S rRNA sequencing analysis, as well as bacterial changes observed in patients with muscular dystrophy. To assess alterations in relative abundance at the phylum and genus levels of bacteria, trends indicating increased or decreased relative abundances were extracted for five and seven major bacterial taxa, respectively. For analyzing changes in relative abundance at the genus and species levels of bacteria, mean values along with their corresponding standard deviations (SD) were directly obtained from 10 included articles. In cases where direct mean values and SD were unavailable, quartile ranges along with maximum and minimum values were extracted from original figures to calculate mean values and SD.

The Newcastle-Ottawa Scale (Zeng et al., 2015) was employed to assess the quality of the literature included in the study. This scale has a maximum score of 7, and studies achieving a total score ≥ 5 are considered as high-quality. Two researchers independently conducted blinded assessments on the included studies, with any discrepancies that arose during this process being resolved through consultation with a third expert.

### 2.5 Meta-analysis

The effect size was calculated using a random-effects, inverse variance weighted model in RevMan 5.3 software. To estimate the mean and standard deviation of median, maximum, and minimum values, we used a previously reported transformation equation assuming mild departure from normal distribution (Hozo et al., 2005). Hedges’ g effect size was computed as the average difference between the sarcopenia group and non-sarcopenia group divided by their combined standard deviation. We assessed heterogeneity of each study using Q-statistics and *I*^2^. Funnel plot was employed for quantitative evaluation of potential publication bias. The significance level was set at *p* < 0.05.

### 2.6 Availability of data

Kindly request the primary author for access to the corresponding data.

## 3 Results

### 3.1 Study selection and characteristics

**Figure 1:**
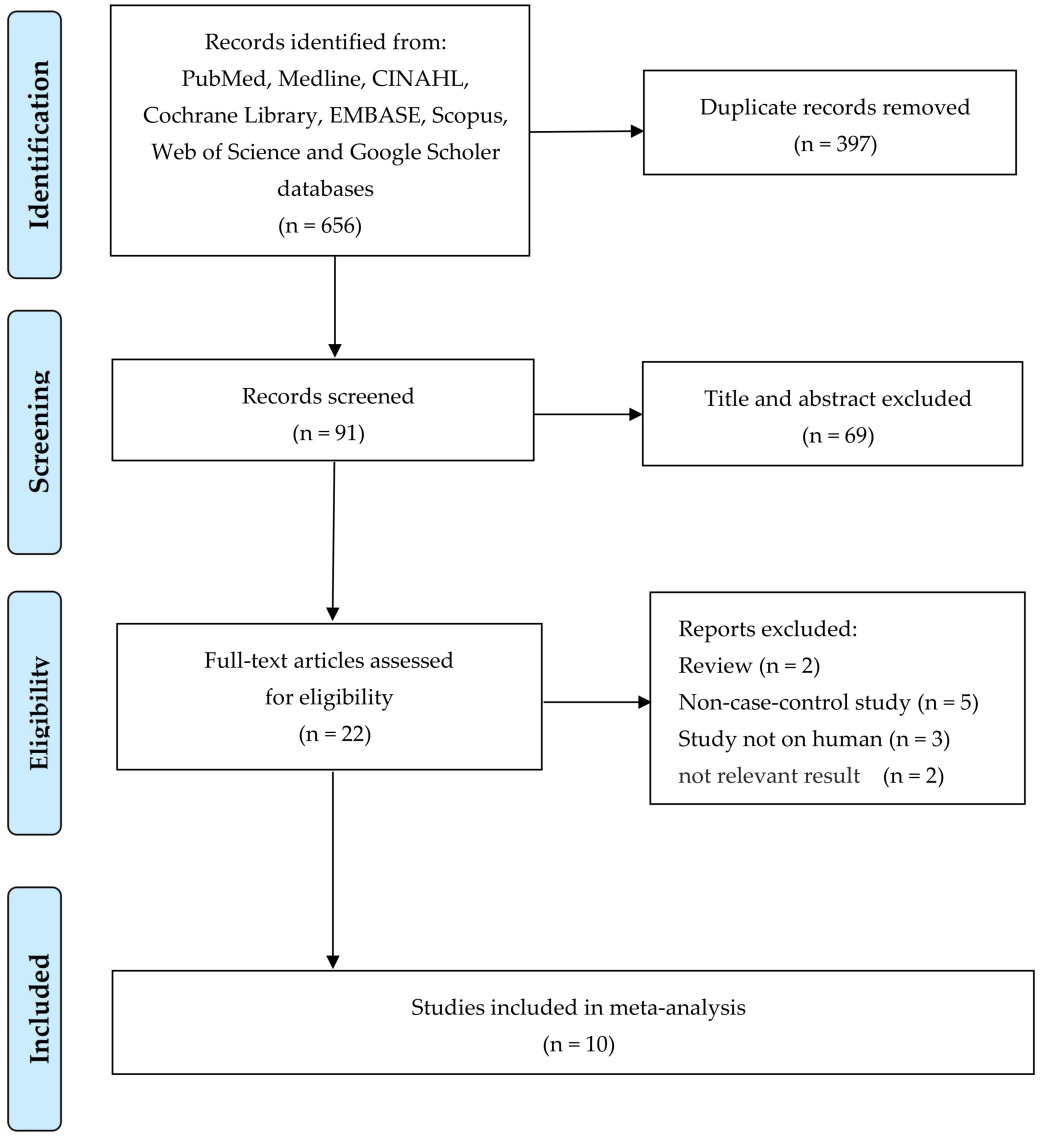
PRISMA flow diagram showing the study selection process.

After conducting a comprehensive literature search and eliminating duplicate articles, we obtained a total of 397 relevant papers. Following the review of titles and abstracts, 16 studies were identified as potentially meeting our inclusion criteria for meta-analysis (Figure 1). Upon meticulous examination of the full texts, 22 studies were subsequently excluded: two were review articles (Liu et al., 2021; Nikkhah et al., 2023), five were non-case–control studies (Jackson et al., 2016; Daily and Park, 2022; Lapauw et al., 2023; Yan et al., 2023; Zhao et al., 2023; Iwasaka et al., 2024), three involved animal experiments (Kim et al., 2022; Lee et al., 2023; Mo et al., 2023), and two were do not mention the diversity of OUT of the microbiota (Chen et al., 2023; Zhou et al., 2023). Consequently, the final meta-analysis comprised 10 remaining articles (Figure 1). All the 10 studies included 869 men and 1,148 women over 60 years old, and 46 people in one study (Yan et al., 2023) did not mention gender. Table 1 provides an overview of the clinical characteristics and demographic features observed in these 10 studies (Kang et al., 2021; Han et al., 2022; Lee et al., 2022; Wang X. et al., 2022; Wang Y. et al., 2022; He et al., 2023; Yan et al., 2023; Zhang et al., 2023; Zhou et al., 2023; Lou et al., 2024). These studies were conducted in Italy (Ticinesi et al., 2020) and China with a collective participation of 421 individuals diagnosed with sarcopenia and 1,642 non-sarcopenia controls (Table 1).

**Table 1:** Characteristics of the studies included.

Study, year	Country	Setting	Age (case/control)	Sample size (case/control)	Gender (case/control)	Gut microbiota assessment	Score
Almohaisen et al. (2022)	Italy	Community	79.50 ± 7.78/72.17 ± 3.70	5/12	Case: (M 1/F 4) Control: (M 2/F 10)	Shotgun metagenomic sequencing	**8**
Han et al. (2022)	China	Hospital	72.30 ± 5.40/70.00 ± 4.20	36/52	Case: (M 20/F 32) Control: (M 8/F 28)	16S rRNA sequencing (V3-V4)	**7**
He et al. (2023)	China	NM	75.13 ± 5.80/69.81 ± 4.60	32/31	Case: (M 7/F 25) Control: (M 12/F 19)	Shotgun metagenomic sequencing	**7**
Lee et al. (2022)	China	Community	66.50 ± 4.60/64.80 ± 3.40	27/33	Case: (M 5/F 22) Control: (M 10/F 23)	16S rRNA sequencing	**8**
Liu et al. (2021)	China	NM	76.45 ± 8.58/68.38 ± 5.79	11/60	Case: (M 4/F 7) Control: (M 27/F 33)	16S rRNA sequencing	**7**
Lou et al. (2024)	China	Hospital	72.50 ± 4.70/71.60 ± 4.50	108/98	Case: (M 66/F 42) Control: (M 61/F 37)	16S rRNA sequencing (V3-V4)	**7**
Wang X. et al. (2022) and Wang Y. et al. (2022)	China	Community	72.20 ± 8.50/62.30 ± 8.50	141/1,276	Case: (M 73/F 68) Control: (M 509/F 767)	Shotgun metagenomic sequencing	**8**
Yan et al. (2023)	China	Community	75.26 ± 7.14/70.26 ± 6.03	17/29	Case: NR Control: NR	16S rRNA sequencing	**8**
Zhang et al. (2023)	China	Hospital	71.21 ± 6.85/70.00 ± 6.67	14/21	Case: (M 7/F 7) Control: (M 18/F 3)	16S rRNA sequencing (V4)	**7**
Zhou et al. (2023)	China	Hospital	88.83 ± 6.30/70.80 ± 8.15	30/30	Case: (M 18/F 12) Control: (M 21/F 9)	16S rRNA sequencing (V3-V4)	**7**

### 3.2 α diversity and β diversity

**Figure 2:**
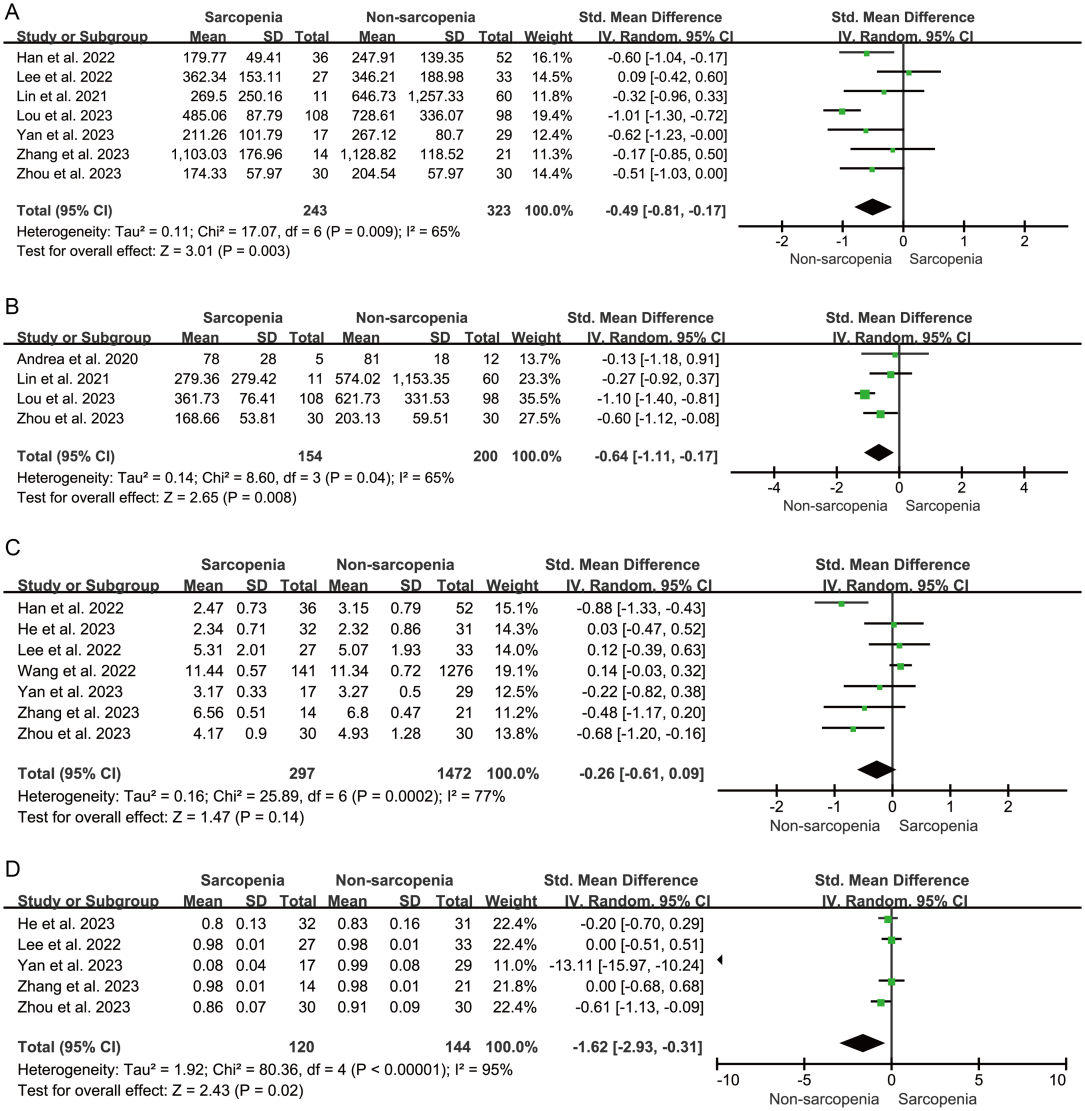
Forest plots of Chao 1 **(A)**, Observed species **(B)**, Shannon **(C)**, and Simpson **(D)** between sarcopenia group and non-sarcopenia group.

The alpha diversity index is a measure of the number of biotic species within a community as well as the relative abundance of biotic species among them. α diversity is mainly Chao1 richness estimator, Shannon diversity index, Simpson diversity index and Observed species were calculated. The results showed that sarcopenia group demonstrated significantly reduced α diversity as indexed by Chao 1 index (*n* = 7, SMD = −0.49, 95% CI: −0.81 to −0.17, *I*^2^ = 65%) (Figure 2A), Observed species index (*n* = 4, SMD = −0.64, 95% CI: −1.11 to −0.17, *I*^2^ = 65%) (Figure 2B), and Simpson index (*n* = 5, SMD = −1.62, 95% CI: −2.93 to −0.31, *I*^2^ = 95%) (Figure 2D). Shannon index showed a decreased trend (*n* = 7, SMD = −0.26, 95% CI: −0.61 to 0.09, *I*^2^ = 77%), although there was no statistical significance (Figure 2C).

The β diversity index is the species diversity between ecosystems, which contains a comparison of taxonomic units. That is to measure the differences between communities. The beta diversity index mainly includes PCoA analysis, and PLS-DA analysis. The evaluation of 13 β diversity indicators were assessed for nine studies, with the exception of one study (Lou et al., 2024) (Table 2). β diversity was significantly different in 8/9 of included studies. The principal coordinate analyses based on Bray–Curtis dissimilarity was most frequently measured, of which one study revealed no significant differences (Ticinesi et al., 2020), while seven studies revealed significant differences between sarcopenia and non-sarcopenia group (Han et al., 2022; Lee et al., 2022; Wang X. et al., 2022; Wang Y. et al., 2022; He et al., 2023; Lee et al., 2023; Yan et al., 2023; Zhang et al., 2023; Zhou et al., 2023). One study applied three methods to assess β diversity (Kang et al., 2021). The three methods are PLS-DA, Unweighted UniFrac distances matrix, and PCoA based on Unweighted UniFrac distances. The results of PLS-DA revealed significant differences, while the result of PCoA based on Unweighted UniFrac distances revealed no significances between sarcopenia and non-sarcopenia group.

**Table 2:** Summary of beta diversity assessments in the included studies.

Study	β diversity	Findings	Statistic value
Almohaisen et al. (2022)	PCoA based on Bray–Curtis dissimilarity	No significant difference in gut microbial composition among S and NS	*p* = 0.360
Han et al. (2022)	PCoA based on Bray–Curtis dissimilarity	A significant difference in gut microbial composition among S and NS	*p* = 0.037
He et al. (2023)	PCoA based on Bray–Curtis dissimilarity	A significant difference in gut microbial composition among S and NS	*p* < 0.050
Lee et al. (2022)	PCoA based on Bray–Curtis dissimilarity	A significant difference in gut microbial composition among S and NS^a^	*p* = 0.049
	PCoA based on Bray–Curtis dissimilarity	No significant difference in gut microbial composition among S and NS^b^	*p* = 0.200
Liu et al. (2021)	PLS-DA	A significant difference in gut microbial composition among S and NS	*p* = 0.0001
	Unweighted UniFrac distances matrix	A significant difference in gut microbial composition among S and NS	NR
	PCoA based on Unweighted UniFrac distances	No significant difference in gut microbial composition among S and NS	*p* = 0.080
Wang X. et al. (2022) and Wang Y. et al. (2022)	PCoA based on Bray–Curtis dissimilarity	A significant difference in gut microbial composition among S and NS^c^	*p* = 0.004
	PCoA based on Bray–Curtis dissimilarity	A significant difference in gut microbial composition among S and NS^d^	*p* = 0.020
Yan et al. (2023)	PCoA based on Bray–Curtis dissimilarity	A significant difference in gut microbial composition among S and NS	*p* < 0.050
Zhang et al. (2023)	PCoA based on Bray–Curtis dissimilarity	A significant difference in gut microbial composition among S and NS	*p* = 0.001
Zhou et al. (2023)	PCoA based on Bray–Curtis dissimilarity	A significant difference in gut microbial composition among S and NS	*p* = 0.004

a At the species level.

b At the ASV level.

c At the genetic level.

d At the species level.

### 3.3 Relative abundance of microbial taxa

**Figure 3:**
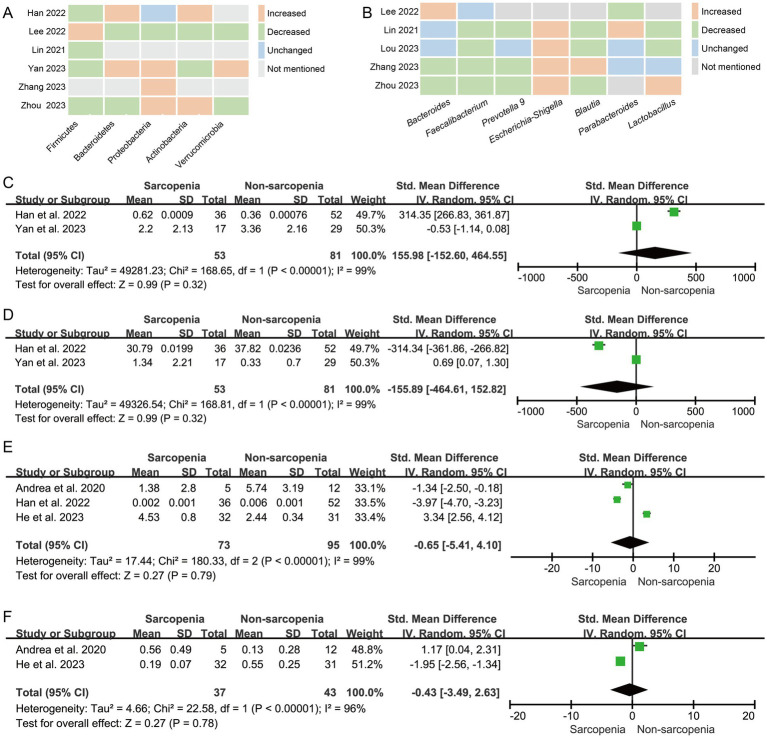
The changes in the relative abundanceof the microbiota in patients with sarcopenia compared with those of non-sarcopenia controls. **(A)** Heatmap analysis at the phylum level; **(B)** Heatmap analysis at the genus level; Forest plots of the relative abundance of **(C)** *Dorea*, **(D)** *Bacteroides*, **(E)** *Faecalibacterium prausnitzii*, and **(F)** *Bifidobacterium longum* between sarcopenia group and non-sarcopenia group.

Six studies were included to analyze the differences in the representative phyla of the microbiome in patients with sarcopenia and non-sarcopenia group. The trend of the relative abundance of phylum Proteobacteria (60%, 3/5) was increased, and the trends of Firmicutes (80%, 4/5) was decreased in the sarcopenia group (Figure 3A).

Seven studies were included to analyze the differences in the representative genera of the microbiome in patients with sarcopenia and non-sarcopenia group. The trend of the relative abundance of genus *Escherichia-Shigella* (100%, 4/4) was increased, and the trends of *Faecalibacterium* (80%, 4/5), *Prevotella 9* (75%, 3/4), and *Blautia* (75%, 3/4) were decreased in the sarcopenia group (Figure 3B).

In the meta-analysis of *Dorea* abundance, Two studies were included. The forest plot indicated that there was no statistically significant difference between sarcopenia group and non-sarcopenia group (*n* = 2, SMD = 155.98, 95% CI: −152.60 to 464.55, *I*^2^ = 99%) (Figure 3C). The results of *Bacteroides* (*n* = 2, SMD = −155.89, 95% CI: −464.61 to 152.82, *I*^2^ = 99%) (Figure 3D), *Faecalibacterium prausnitzii* (*n* = 3, SMD = −0.65, 95% CI: −5.41 to 4.10, *I*^2^ = 99%) (Figure 3E) and *Bifidobacterium longum* (*n* = 2, SMD = −0.43, 95% CI: −3.49 to 2.63, *I*^2^ = 96%) (Figure 3F) were consistent with *Dorea*.

### 3.4 Sensitivity analysis and publication bias

The sensitivity analysis showed that excluding individual studies one by one had no significant impact on the standardized mean difference (SMD), which indicated that the SMD is not influenced by any individual study. Funnel plots (Supplementary Figure S1) indicated a lack of significant publication bias.

## 4 Discussion

In recent years, the hypothesis of gut-muscle axis has been a popular topic of research at home and abroad (Liu et al., 2021). The current study found that the gut microbiota could affect host function in variety of ways and regulate the onset and progression of sarcopenia. For example, Studies have shown that intestinal microbiota could affect skeletal muscle by participating in the regulation of inflammation, immunity, endocrine system and protein synthesis (Li et al., 2022).

To the best of our knowledge, this current meta-analysis represents the first attempt to evaluate α-diversity and β-diversity in individuals diagnosed with sarcopenia. For the result of Shannon, excluding this study due to a significant difference in sample size (2658) compared to other studies (35–87), the findings become statistically significant (*n* = 6, SMD = −0.36, 95% CI: −0.70 to −0.013, *I*^2^ = 61%). Numerous studies have consistently reported a significant reduction in alpha diversity among patients with sarcopenia (Wang X. et al., 2022; Wang Y. et al., 2022; Lou et al., 2024). Additionally, Kang et al.’s investigation demonstrated that older adults exhibiting lower muscle mass exhibited significantly diminishedα diversity within their microbiota community (Kang et al., 2021). These findings align harmoniously with the outcomes of our meta-analysis. It is worth noting that muscle mass typically exhibits a positive correlation with muscle strength (function). Comparing the gut microbiota of women with and without sarcopenia, the women with sarcopenia showed low diversity, which predicted low health status (Sanz et al., 2018), and the reduced diversity of gut microbiota may impair the integrity of the intestinal barrier, allowing harmful substances including lipopolysaccharide to enter the bloodstream, which can not only trigger systemic inflammation, but also induce the up regulation of proinflammatory cytokines, ultimately stimulating protein catabolism and inhibiting muscle synthesis (Grosicki et al., 2018; Ashworth, 2021). Furthermore, this study suggests that there are notable disparities in gut microbiota composition between the sarcopenia group and non-sarcopenia group. Considering beta diversity, further investigation into disparities between healthy controls and individuals with sarcopenia appears necessary due to inconsistent findings.

In the colon of newborn healthy mammals, there is a slightly higher abundance of Proteobacteria, which primarily facilitate oxygen absorption and create a favorable environment for the colonization of obligate anaerobic bacteria. However, in adult healthy mammals, the abundance of Proteobacteria decreases to primarily support the host in maintaining an anaerobic intestinal environment (Bradley and Pollard, 2017). Common gastrointestinal tract bacteria belonging to the family Proteobacteria include *Escherichia coli*, *Salmonella*, *Shigella*, and *Pseudomonas*. The presence of reports suggests that an elevated proportion of Proteobacteria serves as a reliable indicator for dysbiosis in the gut microbiota (Fujisaka et al., 2023). The members of the phylum Proteobacteria are known for their complexity, however, the majority of Proteobacteria found in the gastrointestinal tract are facultative anaerobic and gram-negative bacteria. These bacteria have the ability to produce lipopolysaccharides (LPS) and stimulatory flagellar proteins, which can induce inflammatory reactions, thereby exhibiting a certain degree of pathogenicity. Research has demonstrated that LPS promotes inflammation primarily by entering the bloodstream via the intestinal tract and triggering an inflammatory response. Disruption of the intestinal microecological balance leads to a decrease in beneficial bacteria, increased intestinal permeability, and elevated expression of Gram-negative bacteria, which secrete endotoxins LPS. These endotoxins enter the bloodstream, bind to endotoxin-binding proteins, and are subsequently phagocytosed by macrophages expressing CD14. The activation of CD14 initiates a cascade of cellular metabolic reactions, leading to the secretion of various inflammatory factors from the cell nucleus, such as interleukin-6 (IL-6), tumor necrosis factor-alpha (TNF-α), and gamma interferon. This results in a persistent low-grade chronic inflammatory state within the body. Chronic inflammation further contributes to the development of chronic diseases and accelerates the aging process (Qin et al., 2024).

*Escherichia-Shigella* has been associated with a pro-in flammatory statusand (Zhao et al., 2022), persistent infection with adherent and invasive Escherichia led to chronic and persistent peripheral inflammation (Liang et al., 2022). Cattaneo found a positive correlation between changes in the abundance of *Escherichia-Shigella* and changes in the levels of the pro-inflammatory mole cules IL-6, CXCL2, and NLRP3, the genus Escherichia to induce the production of pro-inflammatory cytokines through NLRP3-dependent mechanism (Cattaneo et al., 2017). Moreover, changes in the abundance of inflammation-related bacteria, such as *Shigella* and *Agathobacter*, were observed in female sarcopenia patients (Norman et al., 2021). Inflammation is thought to underlie various physiological and pathological processes. Age-related chronic low-grade inflammation is one of the important factors of sarcopenia. Macrophages released pro-inflammatory factors, reduced proteosynthesis, and raised protein degradation of the skeletal muscle through a variety of ways (Nardone et al., 2021). Many bacterial pathogens, including Shigella, may increase skeletal muscle damage by provoking inflammasome activity and inducing inflammatory responses (Suzuki et al., 2014). The pathogenic mechanism of *Escherichia-Shigella* involves a series of critical steps: (1) traversing the gastrointestinal barrier to reach the colon; (2) being engulfed by resident colonic macrophages, which subsequently triggers inflammasome activation; (3) the activated inflammasome induces macrophage death, facilitating Shigella’s escape; and (4) invading colonic epithelial cells and spreading to neighboring cells, leading to epithelial cell death, ulceration, increased fluid accumulation in the colon, and the formation of pus, blood, and mucus. It is evident that Shigella’s capacity to evade immune surveillance and escape plays a crucial role in its pathogenicity. Previous research has demonstrated that Shigella employs the type III secretion system (T3SS) to inject effector proteins into host cells, thereby aiding the bacterium in evading host immune defenses. Specifically, natural killer (NK) cells secrete granzyme A, which targets gasdermin B (GSDMB) in epithelial cells, promoting its cleavage and activation. This process enables GSDMB to perforate bacterial cell membranes, resulting in bacterial death. However, wild-type Shigella can counteract this effect by using the effector protein IpaH7.8 to promote the proteasomal degradation of GSDMB, thereby avoiding the bactericidal action of NK cells (Hansen et al., 2021).

Moreover, the supplementation of additional probiotics is recognized as a viable nutritional intervention that can enhance muscle mass and/or function while contributing to the prevention of muscle-degenerative diseases (Liu et al., 2021). Furthermore, Lee et al. observed that probiotics have the potential to enhance muscle mass, handgrip strength (HGS), gait speed, and balance while simultaneously mitigating sarcopenia, physical frailty, and fall incidence in elderly patients with primary osteoporosis (Karim et al., 2022). Another investigation further indicated that male athletes who consumed probiotics exhibited enhancements in muscle mass, strength, and exercise recovery (Przewlocka et al., 2020). The role of the gut microbiota in the development of muscle loss during aging is a crucial area that requires further studies for translation to patients.

### 4.1 Limitations

Despite these interesting findings, our study is not without limitations. Firstly, the results of generalizability to other populations is questionable, because the vast majority of research into only from the two countries. Secondly, many studies are subject to significant bias. Statistically significant heterogeneity was observed among the included studies, which may be due to differences in dietary patterns, geographical background, and disease inclusion criteria (including different treatment regimens, medication doses, duration of disease, age, etc.). Nevertheless, we applied the random-model to estimate the effect sizes to reduce the influences of the heterogeneities on our results. Thirdly, it is important to consider that the utilization of diverse nucleic acid extraction methods and gene sequencing techniques (Table 1) may potentially introduce bias into the obtained results. For example, the differences ofαdiversity between sarcopenia and non-sarcopenia might be greater based on the V3-V4 region than those on the V4 region. However, the limited number of studies (one with V4 region and three with V3-V4 region) impeded us to perform additional analysis. Fourthly, in several studies, we manually extract the necessary data from the histogram, this may lead to another type of deviation. However, the procedure was carried out by two reviewers with full discussion and consensus. Therefore, we reasoned that the direction of statistical significance for between-group comparisons would not be materially affected because we performed this approach uniformly throughout the study. Fifthly, the effects in many occasions were assessed by very few studies and thus the current results should be interpreted with cautions. It merits future research to include more studies to provide stronger evidence on this issue.

## 5 Conclusion

In conclusion, we demonstrated that patients with sarcopenia showed significantly reduced α diversity in gut microbiota, and the composition of gut microbiota was significantly different with that of non-sarcopenia group. We also found more abundance of phylum Proteobacteria and genus *Escherichia-Shigella*, and less abundance of phylum Firmicutes and genus *Faecalibacterium*, *Prevotella 9*, *Blautia* in the sarcopenia group. In the future, a larger cohort study is needed to further examine the differences of gut microbiota in sarcopenia group.

## Data Availability

The datasets presented in this study can be found in online repositories. The names of the repository/repositories and accession number(s) can be found in the article/Supplementary material.

## Supplementary material

Figure S1 Funul plots of Chao 1 **(A)**, Observed species **(B)**, Shannon **(C)**, and Simpson **(D)**, *Dorea* **(E)**, *Bacteroides* **(F)**, *Faecalibacterium prausnitzii* **(G)**, and *Bifidobacterium longum* **(H)**.
