# Correction: Gut microbiota in patients with sarcopenia: a systematic review and meta-analysis

**DOI:** 10.3389/fmicb.2026.1878167

**Published:** 2026-06-12

**Authors:** Guangning Wang, Yujie Li, Huisong Liu, Xinjuan Yu

**Affiliations:** 1 Department of Critical Care Medicine, Qingdao Hospital, University of Health and Rehabilitation Sciences, Qingdao, China; 2 Reproductive Medicine Center, Women and Children's Hospital, Qingdao University, Qingdao, China; 3 Department of Nursing, Qingdao Hospital, University of Health and Rehabilitation Sciences, Qingdao, China; 4 Department of Clinical Research Center, Qingdao Hospital, University of Health and Rehabilitation Sciences, Qingdao, China

**Keywords:** gut microbiota, sarcopenia, musculoskeletal diseases, effects, meta-analysis

An incorrect **Funding** statement was provided.

The correct **Funding** statement reads:

The author(s) declared that financial support was received for this work and/or its publication. This study was supported by the 2024 Qingdao Medical and Health Research Project (Grant No. 2024-WJKY020).

The original version of this article has been updated.

