# Supplementary figures and images for: Gut microbiota in patients with sarcopenia: a systematic review and meta-analysis
Source: Front Microbiol. 2025 Jan 22;16:1513253. doi: 10.3389/fmicb.2025.1513253 (PMC11794218; doi:10.3389/fmicb.2025.1513253)

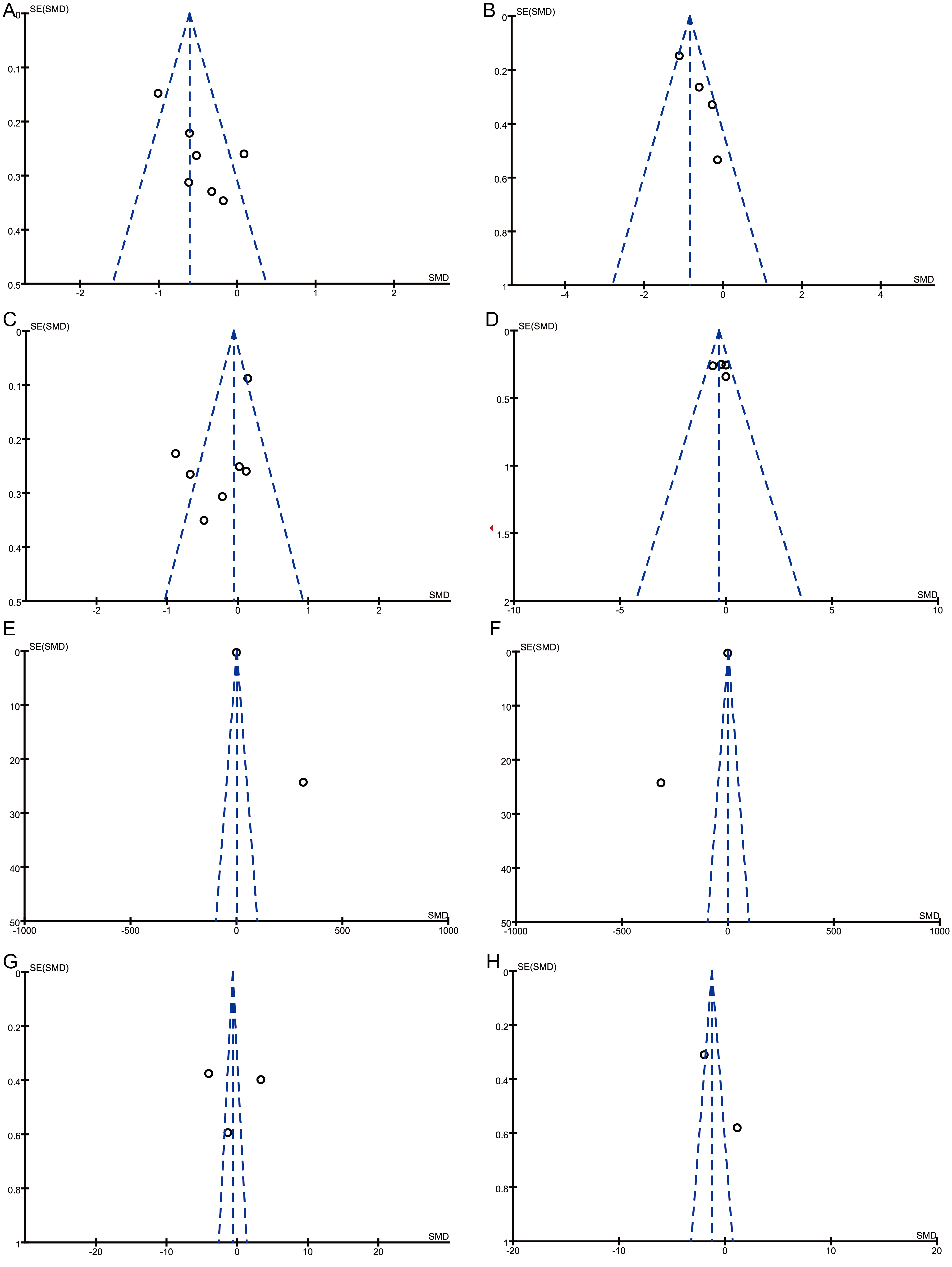

Supplement: Figure S1 — Funul plots of Chao 1 (A), Observed species (B), Shannon (C), and Simpson (D), Dorea (E), Bacteroides (F), Faecalibacterium prausnitzii (G), and Bifidobacterium longum (H). [file Image_1.tif]
